# Characterization and Expression Analysis of Extradiol and Intradiol Dioxygenase of Phenol-Degrading Haloalkaliphilic Bacterial Isolates

**DOI:** 10.1007/s00284-022-02981-8

**Published:** 2022-08-22

**Authors:** Nasser H. Abbas, Afaf Elsayed, Hamdy A. Hassan, Sabha El-Sabbagh, Ashraf F. Elbaz, Hany Khalil

**Affiliations:** 1 Department of Molecular Biology, Genetic Engineering and Biotechnology Research Institute, University of Sadat City, Sādāt, Egypt; 2 Botany and Microbiology Department, Faculty of Science, Menoufia University, Menoufia, Egypt; 3 Department of Microbiology, Genetic Engineering and Biotechnology Research Institute, University of Sadat City, Sādāt, Egypt; 4 Industrial Biotechnology Department, Genetic Engineering and Biotechnology Research Institute, University of Sadat City, Sādāt, Egypt

## Abstract

Haloalkophilic bacteria have a potential advantage as a bioremediation organism of high oil-polluted and industrial wastewater. In the current study, Haloalkaliphilic isolates were obtained from Hamralake, Wadi EL-Natrun, Egypt. The phenotype script, biochemical characters, and sequence analysis of bacterial-16S rRNA were used to identify the bacterial isolates; *Halomonas HA1* and *Marinobacter HA2*. These strains required high concentrations of NaCl to ensure bacterial growth, especially *Halomonas HA1* strain. Notably, both isolates can degrade phenol at optimal pH values, between 8 and 9, with the ability to grow in pH levels up to 11, like what was seen in the *Halomonas HA1* strain. Moreover, both isolates represent two different mechanistic pathways for phenol degradation. *Halomonas HA1* exploits the 1,2 phenol meta-cleavage pathway, while *Marinobacter HA2* uses the 2,3 ortho-cleavage pathway as indicated by universal primers for 1,2 and 2,3 CTD genes. Interestingly, *Marinobacter HA2* isolate eliminated the added phenol within an incubation period of 72 h, while the *Halomonas HA1* isolate invested 96 h in degrading 84% of the same amount of phenol. Phylogenetic analysis of these 1,2 CTD (catechol dioxygenase) sequences clearly showed an evolutionary relationship between 1,2 dioxygenases of both *Halomonadaceae* and *Pseudomonadaceae*. In comparison, 2,3 CTD of *Marinobacter HA2* shared the main domains of the closely related species. Furthermore, semi-quantitative RT-PCR analysis proved the constitutive expression pattern of both dioxygenase genes. These findings provide new isolates of *Halomonas* sp. and *Marinobacter* sp. that can degrade phenol at high salt and pH conditions via two independent mechanisms.

**Supplementary Information:**

The online version contains supplementary material available at 10.1007/s00284-022-02981-8.

## Introduction

Phenol is an important industrial chemical that is utilized as an intermediate substance for chemical products such as xylenols and oil refining [1]. Phenolic compounds constitute one primary source of industrial pollutants because of their toxicity [2]. Accordingly, the phenol-contaminated hypersaline effluent of industrial waste is treated through different chemical protocols [3]. The high expense of such labor and complicated techniques elucidate the need for biological treatments with less economic costs [4]. In this way, there are several reports showed the efficiency of aerobic microbial phenol degradation under hypersaline conditions [5, 6].

Aerobic phenol degradation includes two highly conserved enzyme systems known as intradiol-cleaving and extradiol-cleaving enzymes; both of them use NADH as an electron donor and molecular oxygen to cleave aromatic rings. Another shared requirement for these two enzymatic systems is the using of non-heme iron to establish the functional enzymatic structure required for binding substrate [7].; The intradiol-cleaving enzyme cleaves the bond between the two hydroxyl groups, while the extradiol-cleaving enzyme cleaves the ring between one hydroxyl group and its adjacent non-hydroxylated carbon [8]. In vivo, the formation of catechol is the initial product of monooxygenase oxidation of the phenyl ring; then the oxidative cleavage is processed either through meta-cleavage (generates 2 hydroxymuconicsemialdehyde) or ortho-cleavage (generates muconic acid). The ultimate product of further oxidation is beta-ketoadipate, which enters the tricarboxylic acid cycle [9]. In general, haloalkaliphilic bacteria possess unique adaptation mechanisms to survive and grow under salinity and alkaline pH. These properties make them attractive candidates for fundamental research and biotechnological points of view [10, 11].

In this study, we aimed to isolate and identify phenol degradation haloalkaliphilic bacteria from the Hamra-lake depression in Wadi El-Natrun, located in the Sahara desert, 90 km north-west of Cairo, Egypt. Notable, this alkaline and hypersaline lake aggregate has a pH value between 8.5 and 11 and was considered as hypersaline and alkaline aquatic ecosystem rich in sulfate, chloride, carbonates, and sodium [12].

## Materials and Methods

### Samples and Culture Conditions

Water samples were collected from Hamralake in Wadi El Natrun, Egypt (30° 10′ N, 30° 27′ E), in which the pH is 10.0 and water salinity is 300 g/L. Halophile growth medium (HGM) was prepared as previously described [13], and the pH was adjusted using NaHCO_3_. The media was supplemented with 3 M NaCl and 2.5 mM phenol as a sole carbon and energy source. The water samples were added to HGM media in a ratio of 1:10/v and incubated at 30 °C for 2 weeks. The successive growth media was carried out using 50 µl of diluted culture, which spread on agar plates with the same media. A single colony was inculcated individually on new agar plates with the same growth media for biochemical characterization.

### Phylogenetic Analysis of the 16S Ribosomal RNA Gene

Genomic DNA was extracted from the pure culture using GeneJET Genomic DNA Purification Kit (Thermo Scientific). PCR amplification of the 16S rRNA gene was carried out using Bact 27f (5′-AGAGTTTGATC(A/C)-TGGCTCAG-3′) and Bact 1492r (5′-TACGG(C/T)-ACCTTGTTACGACTT-3′) [14, 15]. According to the manufacturer’s protocols, the amplified products were sequenced using a 3100 Genetic Analyzer (Applied Biosystems) [16]. The obtained sequence of the 16S rRNA gene was compared with the nucleotide sequences collection (nr/nt) database using the BLASTN program and the National Center for Biotechnology Information website (http://blast.ncbi.nlm.nih.gov/Blast.cgi). Based on high-scoring BLAST hits, the phylogenetic tree was performed using the (MEGA 7.0.26) software [17].

### Bacterial Growth and Phenol Degradation

HGM medium amended with 2.5 mM phenol, as a sole carbon source, was used to monitor phenol degradation by bacterial isolates. Different temperatures, NaCl concentration, and pH (using NaHCO_3_ or Na_2_CO_3_) were applied separately to determine the best conditions for bacterial growth in the phenolic condition. The primary culture was prepared by growing bacteria on mineral salt media described above supplemented with 0.3% yeast extract. Then the cells were harvested by spin down at 3000 rpm and 5 °C for 15 min and washed twice with 50 mM phosphate buffer (pH 7), and resuspended in liquid HGM media supplemented with 2.5 mM phenol with an initial optical density of 0.05 (OD_600_). Triplicate samples were centrifuged at regular intervals over an incubation period with optimal environmental conditions, and the OD_600_ was used to measure phenol concentration in parallel. Further, phenol concentrations in the samples were measured using a modified amino antipyrine method [6]. One mL of each sample was centrifuged at 13,000×*g* for 10 min. A volume of 300 µL of the supernatant was added to 6 µL 4-amino-antipyrine (2% w/v) and 6 µL potassium ferricyanide (8% w/v). After an incubation period of 10 min, the solution was mixed with 2 mL of chloroform. The absorbance level of the organic phenol was estimated at 505 nm. Phenol concentration was calculated according to the standard curve with standard phenol concentrations.

### Amplification of Intradiol 1,2 and Extradiol 2,3 Dioxygenase Gene Expression

The presence of intradiol 1,2 CTD was detected in isolated strains using degenerative primer for the highly conserved region (~ 400 bp), cat1 (5′-ACCATCGARGGYCCSCTSTAY-3′) and cat3 (5′-GTTRATCTGGGTGGTSAG-3′) (R = A or G; S = C or G and Y = C or T), as previously described [18]. The detection of extradiol 2,3 CTD was carried out using degenerated primers C23O- F (5′AGG TGW CGTSAT GAA MAA AGG 3′) and C23O- R (5′TYAGGT SAK MAC GGT CAK GAA 3′) (K = G or T; M = A or C and W = A or T), to amplify (~934 bp) of 2,3 CTD gene, as described by Junca and Pieper [19]. PCR mixture was prepared as the following; 10 µl of PCR master mix (Biovision), 50 pmol of each primer, and 100 ng of genomic DNA. The total volume was adjusted to 20 µl using sterile distilled water. The PCR conditions consisted of an initial cycle of 5 min at 95 °C, followed by 30 cycles of denaturation at 94 °C for 1 min, annealing at 50 °C for 30 s, and extension at 72 °C for 1 min. The complete gene sequence of 1,2 CTD of *Halomonas HA1* isolate was obtained using universal primers DOG F (5′-TGACTGTTAAAATTTATGACACCCCTGAAG-3′) and DOG R (5′-TTATGGACGCGCTTGCAGCTC-3′). These primers were deduced depending alignment of high similarities 1,2 CTD genes. The amplification program was conducted in 35 cycles, including 94 °C for 30 s, annealing at 60 °C for1 min, and extension at 72 °C for 1 min. The complete 2,3-cat gene sequence of *Marinobacter HA2* isolate was isolated using C5 and C31 primers designed according to conserved sequence alignment of high similarity 2,3 cat genes. The primers sequence C5 (′5-ATGAAAAAAGGTGTAATGCGTCC-3′) and C31 (′5-GTTCAGYRYVCGRTCGTGG TAG-3′) (V = A, C or G) were used. The amplification program was conducted in 35 cycles, including 94 °C for 30 s, annealing at 58 °C for 30 s, and extension at 72 °C for 1 min. The PCR product was electrophoresed using 1% agarose gel with 0.01% ethidium bromide and visualized with UV illumination. The DNA sequence was carried out using the amplified products and was sequenced using a 3100 Genetic Analyzer (Applied Biosystems) according to the manufacturer’s protocols. The conserved domain analysis was performed using the program of the National Center for Biotechnology Information (NCBI) (https://www.ncbi.nlm.nih.gov/Structure/cdd/cdd.shtml).

### Gene Expression Analysis of 1,2 CTD and 2,3 CTD in *Halomonas *and *Marinobacter* Isolates

Total RNA was purified from 5 ml of bacterial culture grown in a mineral salt media containing phenol (2.5 mM), glucose (5 mM), or the medium contains both of them. RNA was extracted using the GeneJET RNA Purification Kit (Thermo Scientific) and eluted in 50 µl of RNase-free water [20, 21]. According to the manufacturer’s instructions, the extracted RNA was treated with DNase I (Thermo Scientific). First-strand cDNA was carried out using 1 µg total RNA, 2 µl of Maxima Enzyme Mix Reverse Transcriptase (Thermo Scientific), 200 pmol of cat3 gene primer, and 4 µl of the supplied buffer in 20 µl total volume. Serial dilutions of 10^–3^, 10^–4^, and 10^–5^ of cDNA were prepared in sterilized distilled water [22, 23]. For semi-quantitative RT-PCR, 5 µl of each dilution was used in PCR reaction under the previously described conditions for cat1 and cat3 primer (1,2 CTD conserved region of *Halomonas HA1*) and primers C5 and C31 (2,3 CTD gene of *Marinobacter HA2*).

### Statistical Analysis

The Student’s two-tails *t*-test was used to determine the significance of phenol degradation by bacterial isolates. *P* ≤ 0.05 was considered statistically significant (*), while *P* ≤ 0.01 was considered highly significant (**).

## Results

### Identification and Growth Properties of Phenol-Degrading Isolates

The morphological investigation of phenol-degrading isolates revealed the presence of two different isolates; HA1 and HA2 that shared Gram-negative rods with differences in colonies color and shape. The biochemical characters demonstrated a distinguished pattern of urease production, which was negative for HA1 isolate. The full-length 16S rRNA (1500 bp) of both strains were sequenced and deposited under GenBank accession numbers KT223026 (HA1) and KU323642 (HA2). The phylogenetic analysis of HA1 demonstrates a similarity of 98% with the 16S rRNA gene sequence of *H. salifodinae* BC7 while HA2 16S rRNA sequence analysis revealed almost 98.2% similarity with *M. alkaliphilus*. Growth parameters of HA1 isolate showed obligatory salt requirement of 1%, while the bacterial growth can be sustained up to 20% NaCl with optimal growth at 8% NaCl. In addition, the pH tolerance of the strain extended up to pH 11 with optimal growth at pH 9. The optimal growth temperature was 35 °C with the ability to grow up in 50 °C. In contrast, the *Marinobacter HA2* isolate showed a lower tolerance for adverse growth conditions. The best growth layout was obtained at 30 °C, pH 7, and 4% NaCl (Fig S1).

### Phenol Removal Assay

**Fig. 1:**
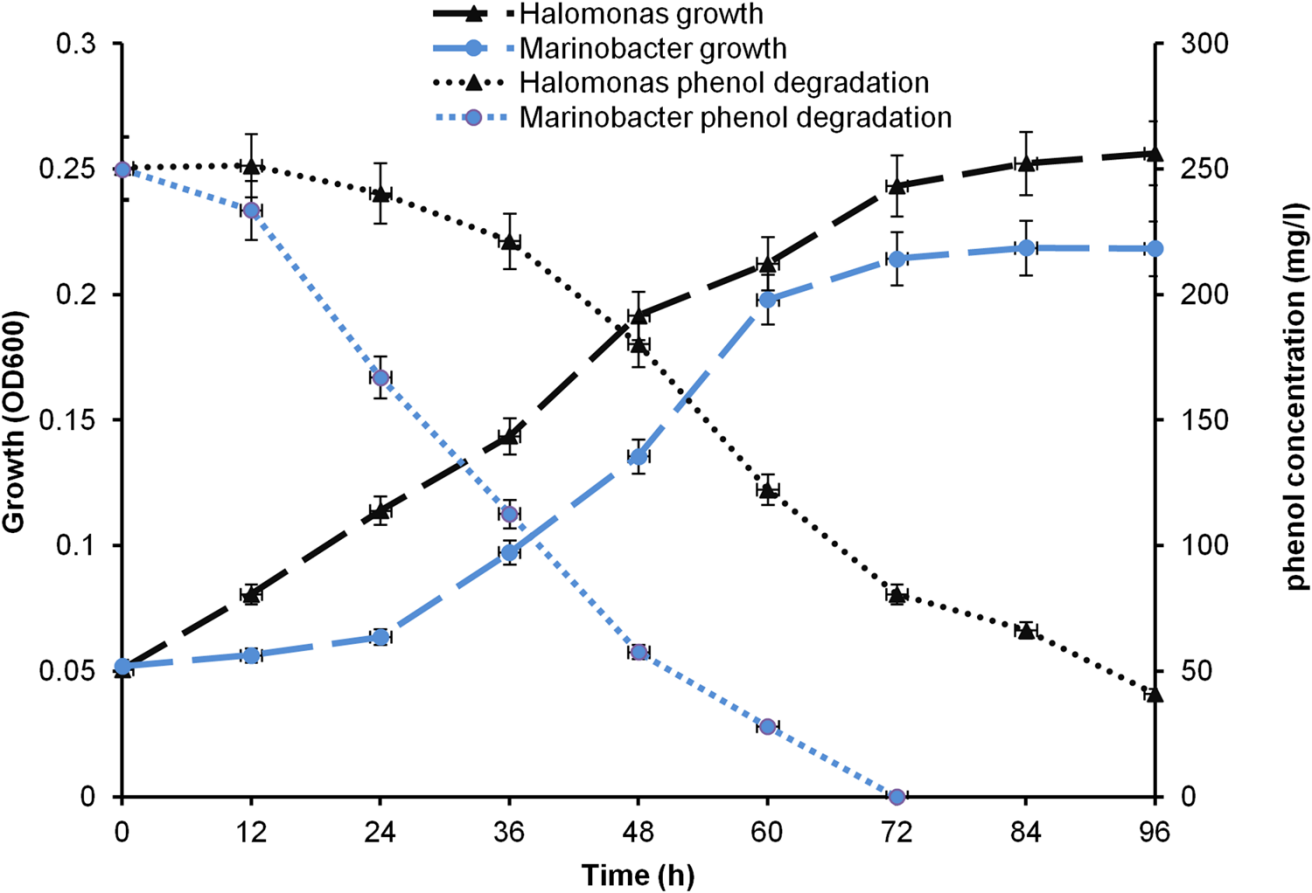
Biodegradation of phenol and growth curve of *Halomonas HA1* and *Marinobacter HA2* bacteria on phenol as the sole carbon source

Both isolates were grown in HGM media supplemented with 2.5 mM phenol as the only carbon source. The incubation temperature, salt concentration, and pH were adjusted independently for each isolate according to its optimal growth conditions. *Halomonas HA1* was grown at a temperature of 35 °C, pH 9, and 10% NaCl, while the optimal growth conditions for *Marinobacter HA*2 included a temperature of 30 °C, pH 7.5, and 4% NaCl. A sterile media supplemented with phenol and bacterial culture without phenol was severed as control. Both types of controls show a steady record of phenol content and absence of growth respectively. *Marinobacter HA2* strain completely degraded the added phenol in 72 h. In comparison, the *Halomonas HA1* isolate was able to degrade only 70% of phenol by the same period. The correlated bacterial cell growth (i.e., OD at 660 nm) was 0.22 and 0.24 for *Marinobacter HA2* and *Halomonas HA1,* respectively (Fig. 1). Statistically, phenol degradation significantly increased in a time-dependent manner of both bacterial growths; however, it showed high significant values by *Marinobacter* isolate in a shorter time (Tables 1 and 2).

**Table 1:** Statistical analysis reveals calculated *P* values of derivative phenol concentration by *Halomonas* isolate

Time hrs O.D	0	12	24	36	48	60	72	84	96	Phenol concentrate-ion (Mg/L)
0.05									0.01**	40
0.06								0.01**		67
0.07							0.01**			81
0.12						0.01**				122
0.17					0.01**					181
0.21				0.02*						220
0.23			0.03*							241
0.25	0.95	0.65								250

*O.D* Optical density, *Statistically significant value, **Highly significant value

**Table 2:** Statistical analysis reveals calculated *P* values of derivative phenol concentration by *Marinobacter* isolate

Time hrs O.D	0	12	24	36	48	60	72	84	96	Phenol concentrate-ion (Mg/L)
0.00							0.001**			0
0.025						0.001**				30
0.05					0.01**					62
0.09				0.01**						120
0.17			0.01**							173
0.23		0.02*								235
0.25	0.8									250

*Statistically significant value, **Highly significant value

### Analysis of the Catabolic Genes

**Fig. 2:**
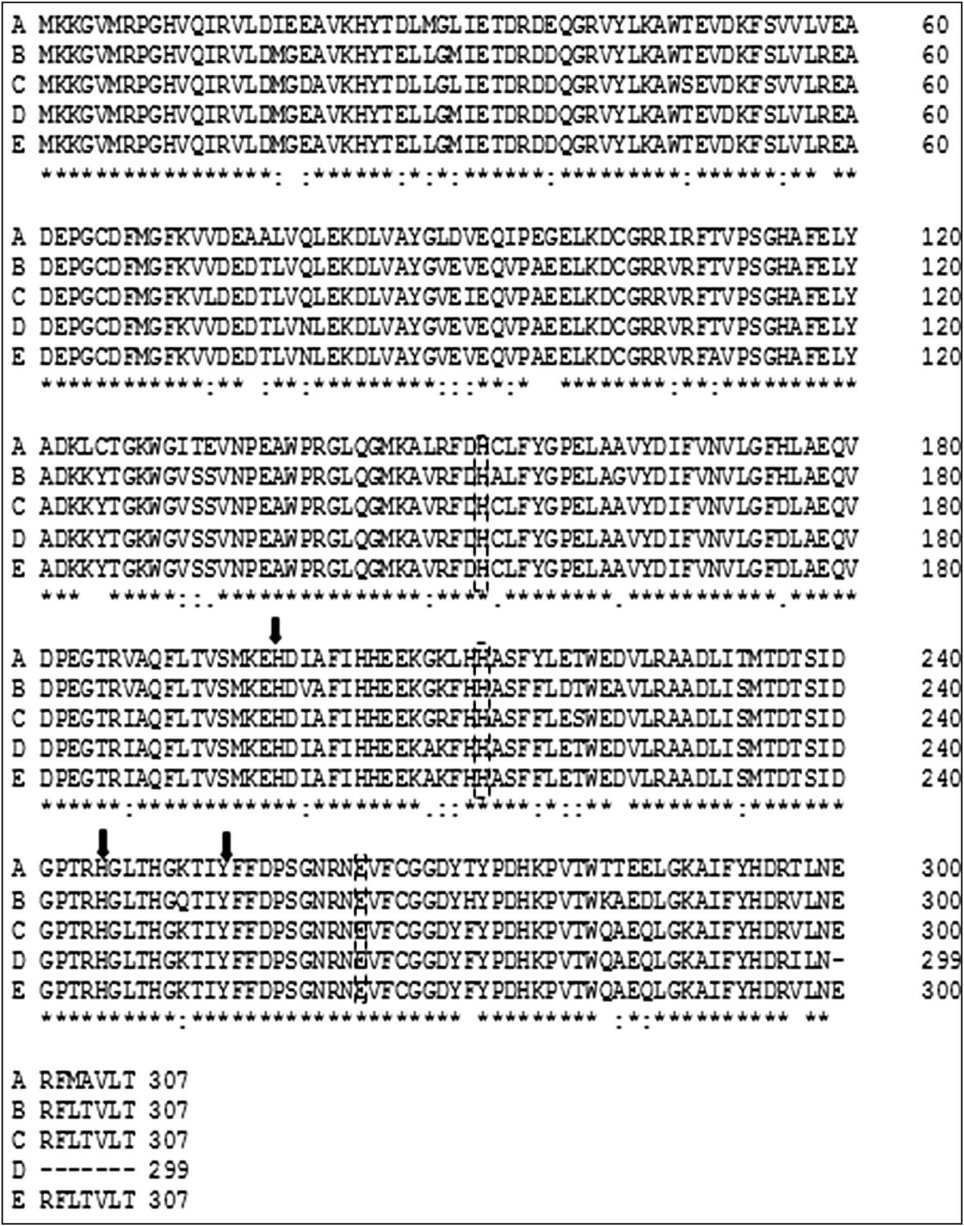
Multiple sequence alignment of conserved 1,2 CTD protein domain. Residues in boxes show the lipid-binding sites. Residues with arrows show the active sites. Residues in discontinuous boxes indicate the Fe-ligands. Bacterial names are indicated in letters as demonstrated: **A** *A. lwoffii* K24 (AAC46228.1), **B** *H. pacifica* (WP_146800704.1), **C** *H. lutea* (WP_019020768.1), **D** *C. salexigens* (WP_110062294.1), **E** *Halomonas* HA1 (AMY26491.1), **F** *P. putida* (WP_020190774), **G** *Pseudomonas* sp. (WP_020190774.1)

**Fig. 3:**
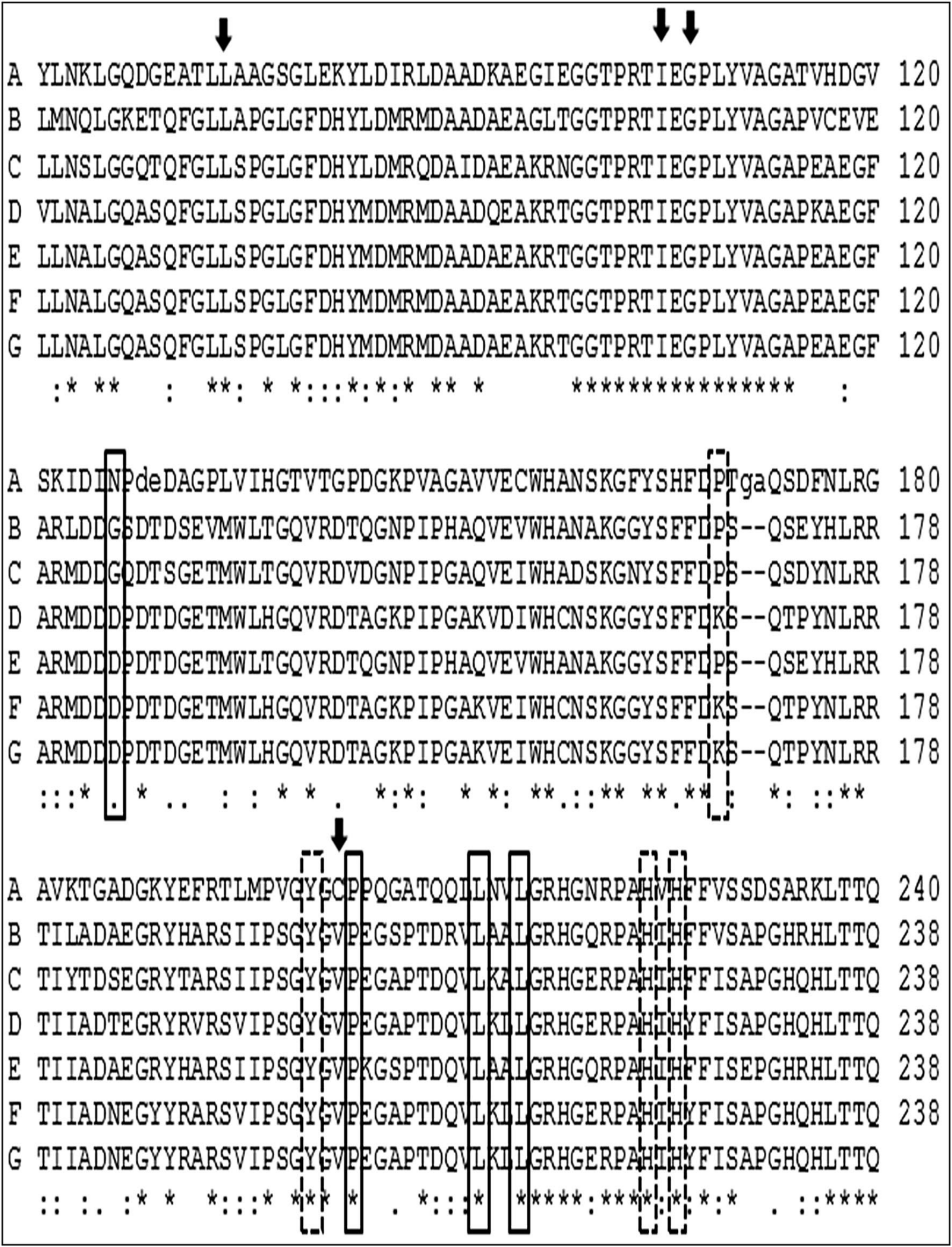
Multiple sequence alignment of conserved 2, 3 CTD protein domain. Residues with arrows show the active sites. Residues in discontinuous boxes indicate the Fe-ligands. Bacterial names are noted in letters as demonstrated: **A** *P. putida* (gbAAQ89675.1), **B** P. stutzeri (gbCAD62376.1), **C** G. bacterium (PKM02781.1), **D** Marinobacter sp. HA02 (gbAMY26490.1), **E** M. excellens (gbKXO08936.1)

Universal primers for the 1,2 CTD gene were used to isolate a gene sequence encoding for 303 amino acid residues that was submitted to the GenBank under accession number AMY26491.1. The amino acid sequence of isolated 1,2 CTD demonstrates the main characters of the conserved 1,2 CTD domains. The conserved domain of substrate binding sites of Leu 73, Ile 105, and Gly 107 showed high corresponding to all intradiol dioxygenases. Other similarities to halophilic 1,2 CTD include Val 200 replacement of the Tyr 200 residue and the presence of Gly 77 instead of Ala as previously reported [18]. The presence of nonheme Fe binding residues Tyr 164, Tyr 198, His 222, and His 224 suggested the trigonal bipyramidal geometry, which is indistinguishable from other intradio dioxygenases structures that were considered by crystallographic examines [24]. The dendrogram of halomonas 1,2-CTD databases indicates that our isolate 1,2 CTD did not share the common ancestor with the most selected *Halomonas* genera. The sequence identity is closer to 1,2-CTD of *H. heilongjiangensis* and *H. pacifica* (Figure S2 and S3). The protein identity was 79% and 76% with 1,2-CTD of *H. heilongjiangensis* and *H. pacifica*. *Marinobacter* 2,3 CTD sequence with 299 amino acids was obtained using the degenerative primers set and submitted to the GenBank under accession number AMY26490.1 (Fig. 2). The sequence with 299 amino acids of *Marinobacter* 2,3 CTD was obtained using the degenerative primers set. The identified protein shares the main conservative domains with other extradiol dioxygenases. As shown in Fig. 3, the Fe II binding sites were recognized in residues of His 153, His 214, and Glu 265. Substrate binding residues were identified as His 246 and Tyr 255 (45). The sequence showed closer evolutionary relations with the sequences of *M. excellens* and *M.* shengliensis with protein identity of 99% with 2,3 CTD protein of *M. shengliensis* and 93% with *M. hydrocarbonoclasticus* 2,3 CTD protein (Figure S4 and S5).

### Transcription Analysis

**Fig. 4:**
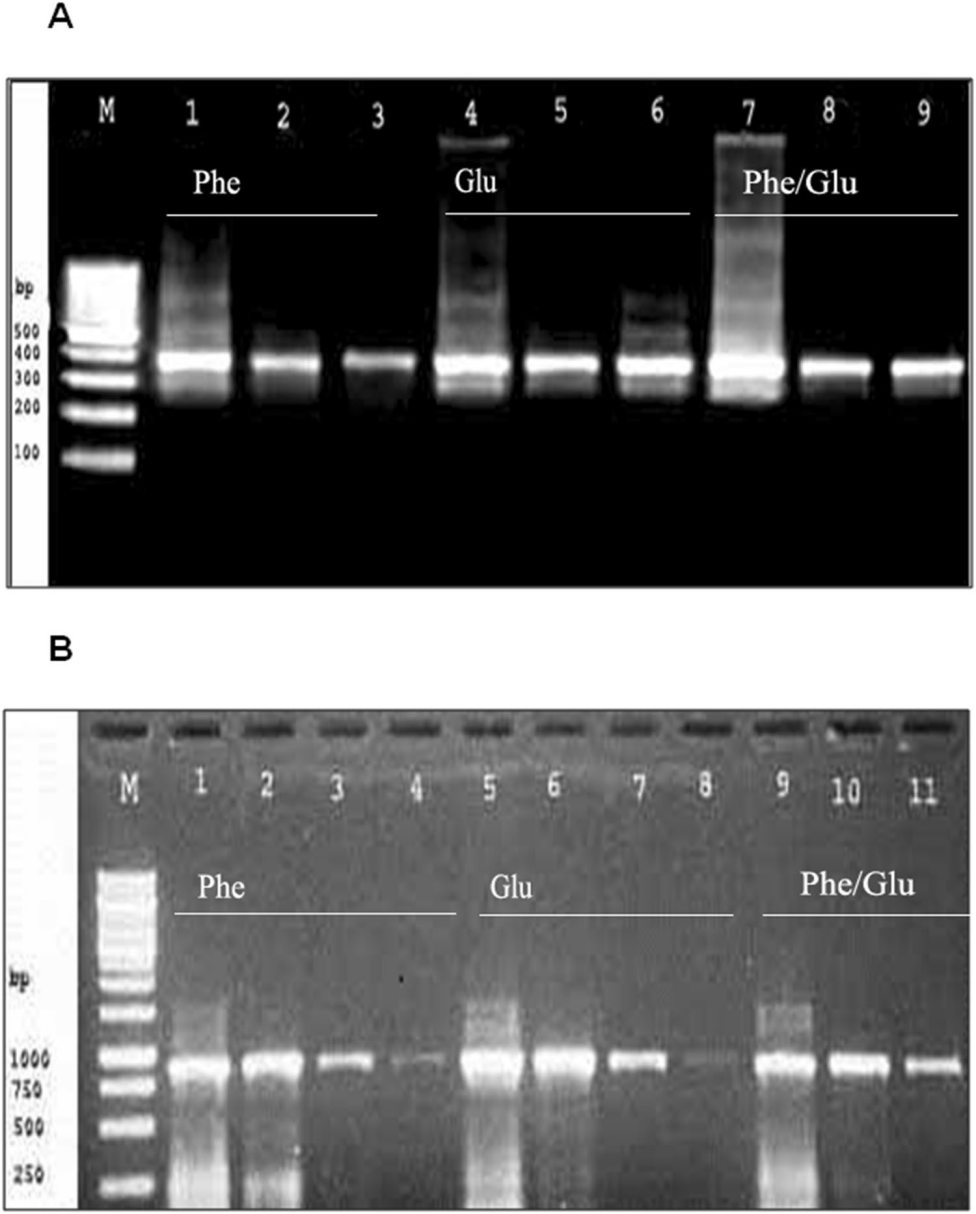
Semi-quantitative RT-PCR detecting the expression pattern. **A** *Halomonas HA1* 1,2 CDG gene in the presence of phenol (1–3) or glucose (4–6) as a sole carbon source and phenol with glucose (7–9). (M) is 100 bp DNA marker. **B** *Marinobacter expression* 2, 3 CDG gene in the presence of phenol (1–4) or glucose (5–8) as a sole carbon source and phenol with glucose (9–11). (M) is a 1 Kb DNA marker

RNA was isolated from a bacterial culture grown on a minimal saline media supplemented with either phenol or glucose as a sole carbon source, or media supplemented with both of them. Primers for the conservative domain of 1,2-CTD *Halomonas sp*. and 2,3-CTD *Marinobacter sp.* were used for RT-PCR. As shown in Fig. 4A and B, an amplified fragment of about 400 bp of the 1,2 CTD gene and 934 bp of 2,3 CTD were obtained with all used carbon sources. This result showed a constitutive gene expression pattern.

## Discussion

Both genera of *Halomonas* and *Marinobacter* contain several members of haloalkaliphilic species, and many of them are known for their ability to degrade aromatic compounds at high pH [25, 26]. Growth parameters optimization for isolated strains demonstrates higher level of stress tolerance potential for *Halomonas HA1* isolate. The high tolerance for salt concentration is a common attribute in this family which may refer to a particular proton translocation system in which NaCl used for glucose adsorption [27]. A special ectoine expression system is another mechanism by which some *Halomonase sp*. can be adapted at a high salt concentration [28]. Under aerobic conditions, phenol degradation pathway is introduced by the action of monooxygenase enzymes and leads to catechol formation. The action of dioxygenases in the dearomatization, the latter catechol pathway includes undergoing ortho-, meta-, or para-cleavage. Ortho-cleavage is catalyzed by catechol 1,2-dioxygenase (intradiol-type dioxygenases) using Fe(II) as a cofactor, which is known as the β-ketoadipate pathway [29]. The meta-cleavage is catalyzed by 2,3-dioxygenase (extradiol-type dioxygenase), using Fe(III) as a cofactor [30]. The metagenomic analysis of the phenol degradation isolates revealed the involvement of either 1,2 CTD or 2,3 CTD in the catabolism of aromatic compounds in positive strains [31, 32]. However, other studies claim that *P. putida* has both cleavage pathways as reported by Basak et al., [33]. In the same ecosystem of Hamra Lake, the two isolates represent two different pathways for phenol degradation; *Halomonas HA1* exploits the 1,2 phenol meta-cleavage pathway while *Marinobacter HA2* uses the 2,3 ortho-cleavage pathway as indicated by universal primers for 1,2 and 2,3 CTD genes. *Marinobacter HA2* isolate can eliminate the added phenol within an incubation period of 72 h. In comparison, *Halomonas HA1* isolate required 96 h to degrade 84% of the same amount of phenol. The presence of the phenol meta-cleavage pathway of *Marinbacter HA2* could interpret the variation of the phenol degradation rate of both strains. The functionality of the 2,3 catechol dioxygenase pathway in phenol degradation may clarify the gene outspread among microbial isolates in environments of phenol-contaminated sediments [34]. According to the ecological advantage, the *Halomonas HA1* isolate showed more growth potential under stress conditions. *Halomonas* genus was represented in the isolated phenol decomposer consortium [35]. The bioremediation ability of a halophilic bacteria *H. organivorans* was proved to catabolize different concentrations of low molecular weight aromatic compounds at 10% NaCl concentration [36]. The phylogenetic-related strain *H. salina* showed phenol degradation efficiency of 66% at 10% NaCl. The catechol 1,2 CTD gene sequences alignment in Fig. 2 exhibits a high degree of divergence among the selected *Halomonas sp*..The 1,2-CTD of *Halomonas HA1* showed segregation in a distinct cluster including the *p. putida* gene. The bacterial phylogenetic estimation based on the 1,2 CTD sequence was previously reported [37, 38]. In this context, our isolates have two different phylogenetic statements based on its 16S rRNA or 1,2 CTD gene sequence. This evidence could indicate the separate evolutionary origin of the isolated 1,2 CTD genes. Many reports demonstrated the role of transposons in the evolution of bacterial dioxygenases [26, 39]. Compared with 16S rRNA, the dioxygenase gene sequence provides an acceptable method for interspecies differentiation among the bacterial genera [37, 38]. A degenerative primer set was used to obtain 299 amino acids sequences of *Marinobacter* 2,3 CTD. The identified protein shares the main conservative domains with other extradiol dioxygenases. The isolated sequence shows high similarity with taxonomically related *M. excellens* and other members of *Microbacteriaceae,* as shown in Fig. 3. In comparison to intradiol 1,2 CTD enzymes, the extradiol 2,3 CTD enzymes show a longer stretch of conserved domains. The expression analysis of *Halomonas HA1* 1,2 CTD and *Marinobacter HA2* 2,3 CTD showed a constitutive gene expression pattern. Many phenol-degrading operons in *proteobacteria* expression are activated by inducers substances of targeted pathway [40, 41]. Phenol and benzoate are the common inducers of 1,2 catechol dioxygenases [42, 43]. The 2,3 meta-cleavage dioxygenase mechanism of *P. pseudoalcaligenes* is mainly utilized with phenol and salicylate inducers [44]. In some cases, both constitutive and induced expression patterns are exhibited by different dioxygenase genes in the same strain [45]. The constituent activation of xenobiotic degrading genes could be a consequence of the presence of an internal promoter that can alter the natural induction of these clusters. The alternation of the transcriptional aspect of phenol-degrading clusters could be an evolutionary advantage by which bacteria can adapt to xenobiotic polluted environments [46]. Evidence indicated the role of class I transposons in Patchwork Assembly of 3-chloro-catechol degradation cluster in *P. stutzeri* [47].

## Conclusion

Phenol is a common industrial pollutant, and its accumulation in the soil causes a severe threat to underground water. There is an urgent need to isolate phenol microbial decomposers that are adapted for high salt and alkaline conditions of industrial wastes. The information about gene structure and expression is necessary to understand degrader gene evolution and regulation. We sought to isolate and identify new isolates of phenol degradation bacteria in the present work. Here two bacterial species, *Halomonas* *HA1* and *Marinobacter HA2,* were isolated from Hamralake in Wadi El Natrun, Egypt. These isolates can degrade phenol at high salt and pH conditions. The two strains proved to follow different strategies for phenol degradation. A New 1,2dioxygenase enzyme has been isolated from *Halomonas* isolate, and its sequence analysis showed an interesting evolutionary intermediate linkage. However,* Marinobacter* isolate revealed the 2,3 catechol dioxygenase activity. Semi-quantitative RT-PCR demonstrated that the expression of both dioxygenases in different isolates was constitutive and not induced by phenol.

## Supplementary Information

Below is the link to the electronic supplementary material.

Supplementary file1 (DOCX 1395 KB)

## Data Availability

All data are included in the manuscript.
