# Supplementary material for: Characterization and Expression Analysis of Extradiol and Intradiol Dioxygenase of Phenol-Degrading Haloalkaliphilic Bacterial Isolates
Source: Curr Microbiol. 2022 Aug 22;79(10):294. doi: 10.1007/s00284-022-02981-8 (PMC9393131; doi:10.1007/s00284-022-02981-8)
Supplement: Supplementary file 1 — Supplementary file1 (DOCX 1395 KB) [file 284_2022_2981_MOESM1_ESM.docx]

**Supplementary data**


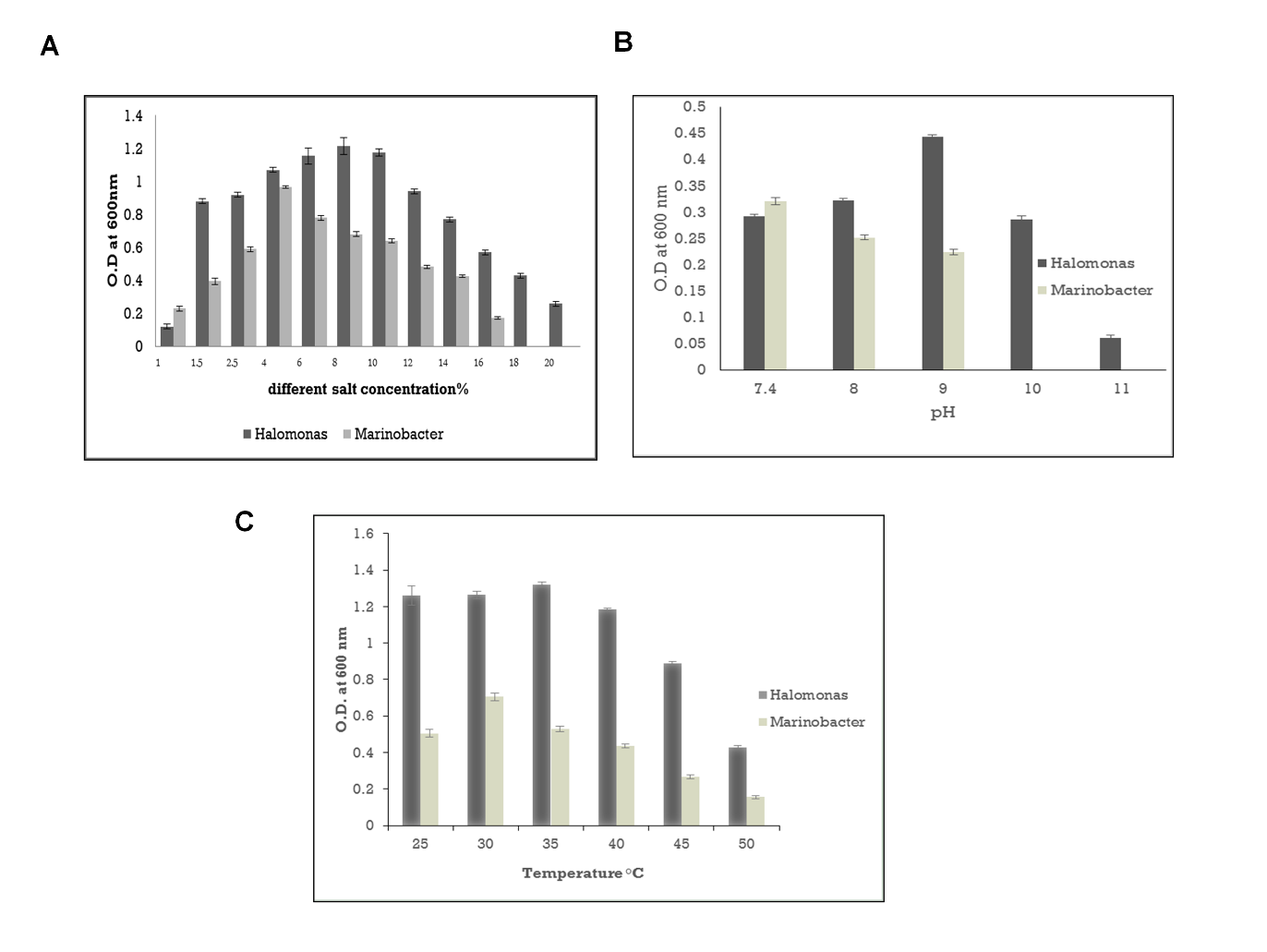


**Figure S1: Growth rate of bacterial isolates in different environmental conditions.** (A) Growth rate in different salt concentrations. (B) Growth rate connected with different pH. (C) Growth rate in different temperature condition.


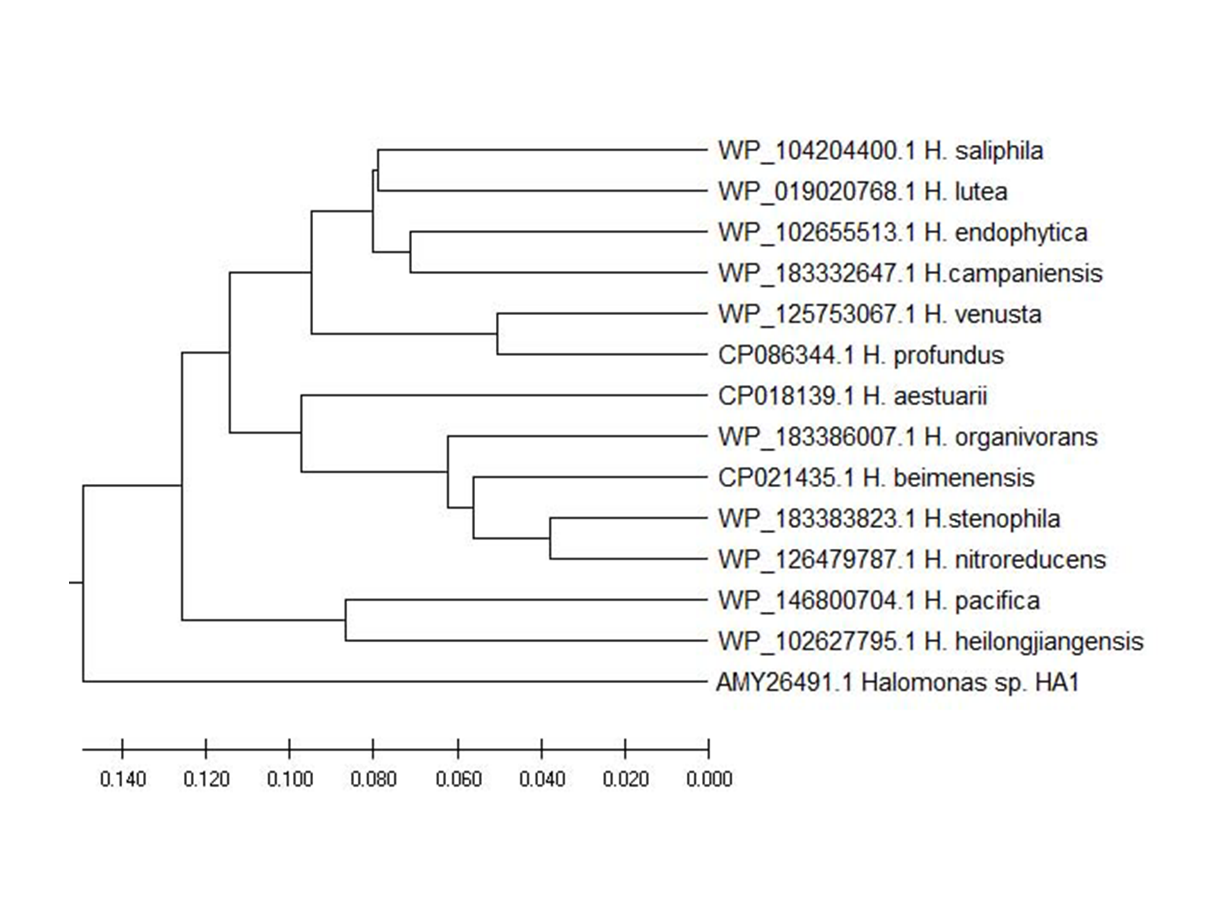


**Figure S2: Phylogenetic tree of 303 aa residues of 1,2 CTD enzyme from *Halomonas HA1* compared to other 1,2 CTD enzymes from related bacteria.** The trees are constructed using MEGA software and the UPGMA method. Branch lengths are proportional to the interfered phylogenetic distances.


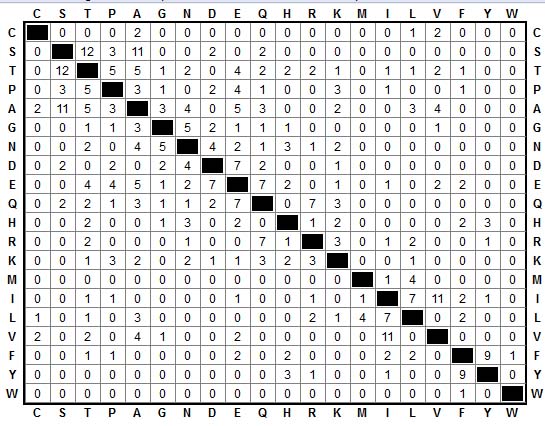


**Figure S3:** Residue substitution analysis of 1,2 catechole alignment using clustalW (Slow/accurate, Gonnet).


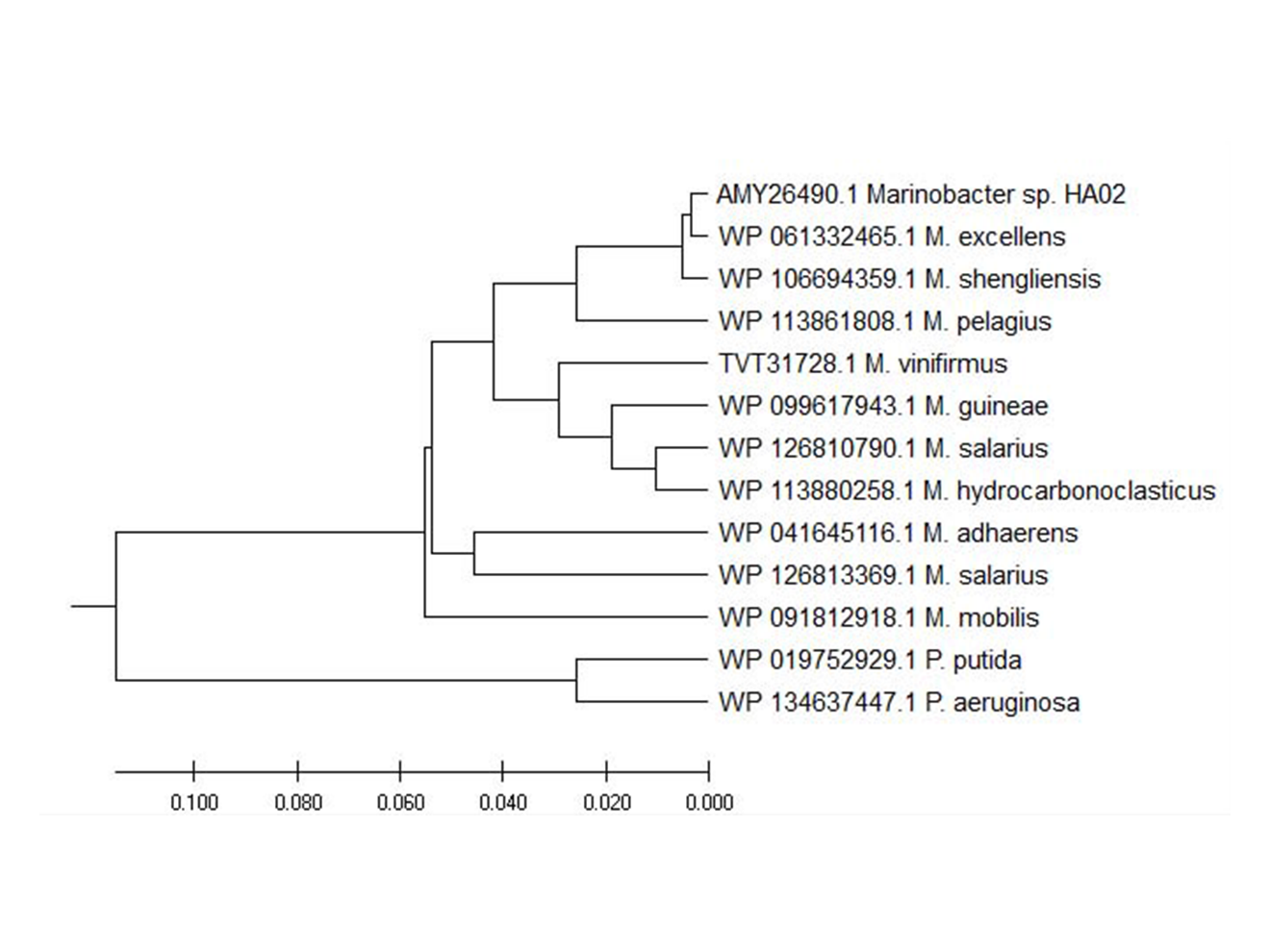


**Figure S4: Phylogenetic tree of 299 aa residues of 2,3 CTD enzyme from *Marinobacter HA2* in comparison with other related family.** The trees are constructed using MEGA software and the UPGMA method. Branch lengths are proportional to the interfered phylogenetic distances.


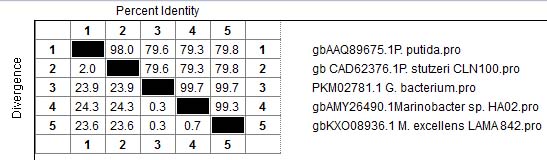


**Figure S5:** Pair distances analysis of 2,3 catechole CDD alignment using clustalW (Slow/accurate, Gonnet).
